# Antibiotic Usage for Treatment of Acute Upper Respiratory Tract Infections in Children in Lithuania from 2018 to 2022

**DOI:** 10.3390/antibiotics14030310

**Published:** 2025-03-17

**Authors:** Tadas Alčauskas, Kristina Garuolienė, Sigita Burokienė

**Affiliations:** 1 Faculty of Medicine, Vilnius University, M. K. Ciurlionio 21, LT-03101 Vilnius, Lithuania; 2 Farmacy and Farmacology Center, Institute of Biomedical Sciences, Faculty of Medicine, Vilnius University, M. K. Ciurlionio 21, LT-03101 Vilnius, Lithuania; kristina.garuoliene@mf.vu.lt; 3 Clinic of Children’s Diseases, Institute of Clinical Medicine, Faculty of Medicine, Vilnius University, M. K. Ciurlionio 21, LT-03101 Vilnius, Lithuania

**Keywords:** acute upper respiratory tract infections, antibiotics, children

## Abstract

**Background/Objectives**: Acute upper respiratory tract infections (URIs) are defined as inflammatory diseases of the nose, sinuses, pharynx, larynx, or trachea. They are common in children. The prescription of antibiotics for the treatment of URIs became a relevant theme in the scientific literature in recent decades. One of the most important ways to deal with increasing antimicrobial resistance is rational antibiotic therapy. This study aimed to evaluate the tendencies of antibiotic prescribing practices for Lithuanian children with URIs from 2018 to 2022. We describe how many children with URIs were prescribed antibiotics, which antibiotics were used, and whether prescribing practices meet national guidelines. **Methods**: Secondary data, which were used in this observational study, were collected from the Lithuanian Compulsory Health Insurance Fund (CHIF) electronic records. The study population consisted of children aged between 0 and 18 years who visited their primary care doctors (pediatricians or family doctors) between January 2018 and December 2022 and were prescribed antibiotics for the treatment of URIs. **Results**: Between 2018 and 2022, there were 445,328 visits reported when antibiotics, which belong to the J01 group according to the Anatomical Therapeutic Chemical Classification (ATC), were prescribed to children aged 0–18. In more than half of the visits (51.70%), children aged 0–5 were consulted. Penicillins were mostly prescribed for the treatment of acute nasopharyngitis. Macrolides were mostly used to treat acute laryngitis and tracheitis. Of all penicillin-class antibiotics, the most popular choice was amoxycillin. The primary choice of cephalosporin was cefadroxil, and the primary choice of macrolide was clarithromycin. **Conclusions**: During the period of 2018–2022, the number of prescriptions for antibiotics for URTIs decreased, but prescriptions for penicillin-class antibiotics increased in a relative manner. The most common diagnoses during these visits were acute tonsillitis and acute pharyngitis, and most antibiotic prescriptions were for children in the 0–5 age group. If Lithuania’s National Recommendations on the Rational Use of Antibiotics were implemented during the analyzed period, the prescribing tendencies would not meet them.

## 1. Introduction

Acute upper respiratory tract infections (URIs) are defined as inflammatory diseases of the nose, sinuses, pharynx, larynx, or trachea [1]. URIs are common in children. According to the Lithuanian Institute of Hygiene, the incidence rate of URIs in 2022 was 511.23 cases/1000 children in Lithuania [2]. It is calculated that on average, children get sick with URIs 5 times a year; however, 10% of children get infected with URIs 10 times a year or more [3].

URIs are usually caused by viruses, while bacteria are responsible for only about 10% of cases [4]. Because of their viral nature, these infections often resolve without the usage of specific drugs or require only symptomatic treatment. Nonetheless, the prescription of antibiotics for the treatment of URIs became a relevant theme in the scientific literature in recent decades [4,5,6]. Inappropriate antibiotic prescribing practices may cause a lot of negative consequences, such as a decrease in microbiota diversity, an increased risk of developing atopic diseases, obesity, inflammatory bowel diseases, etc. [6,7,8,9]. In addition, frequent usage of antibiotics is directly connected to the emerging antimicrobial resistance phenomenon.

In 2019, the World Health Organization (WHO) declared antimicrobial resistance as one of the ten threats to global health [10]. The dangerous nature of this phenomenon can be illustrated by the fact that in 2015, about 30,000 people died in the European Union due to antimicrobial resistance, and 874,541 disability-adjusted life years were lost [11]. Solving this global problem requires a holistic approach [12]. One of the most important ways to deal with increasing antimicrobial resistance is rational antibiotic therapy. Appropriate antibiotic prescribing practices include many aspects: choosing the optimal drug, the effective dose, and the shortest recommended treatment duration, as well as providing the patient and their family with all required information about the treatment [13,14]. Rational prescribing of antibiotics is especially difficult for children because of their inability to formulate precise complaints [15,16,17].

One of the cornerstones of the fight against the inappropriate use of antibiotics in the treatment of URIs is a country-wide situation analysis. Analyzing which antibiotics are prescribed for which diagnoses helps to identify key areas of inappropriate antibiotic prescribing practices and to identify under-prescribing or, conversely, over-prescribing of antibiotics. National electronic systems that record the prescription of medicines can help to achieve this goal.

This study aimed to evaluate the tendencies of antibiotic prescribing practices for Lithuanian children with URIs from 2018 to 2022. We describe how many children with URIs were prescribed antibiotics, which antibiotics were used, and whether or not prescribing practices meet the national guidelines. To achieve this, we requested raw data from the Lithuanian Compulsory Health Insurance Fund (CHIF), which records information on all medicines prescribed in Lithuania, which we processed using statistical methods, and the results are presented in this study.

## 2. Results

**Figure 1:**
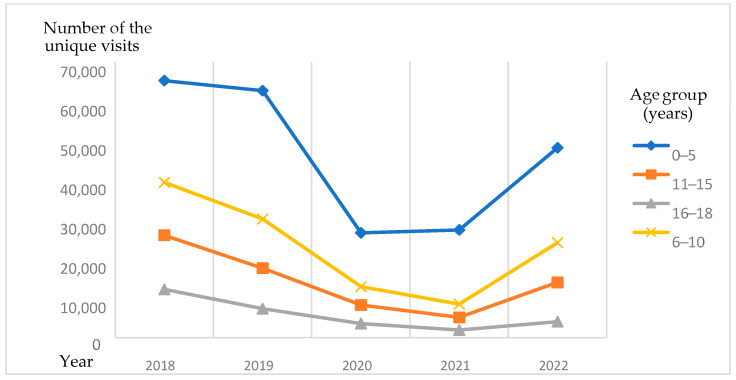
The number of unique visits during the 2018–2022 period.

Between 2018 and 2022, there were 445,328 visits reported when antibiotics, which belong to J0_ group in ATC, were prescribed to children aged 0–18. In more than half of the visits (51.70%), children aged 0–5 were consulted. About a quarter of the remaining visits (25.83%) consisted of children aged 6–10. Other visits were made by children aged 11–15 and 16–18 (15.94% and 6.52% accordingly) (Table 1 for absolute numbers; Figure 1 for tendency visualization).

To contextualize antibiotic prescribing trends in Lithuania in the 2018–2022 period, the number of unique antibiotic prescribing visits per capita in Lithuania in the relevant year in the relevant age group was counted (Table 2).

During the first four years of the analyzed period, the number of visits when antibiotics were prescribed significantly decreased: there were 142,929 visits in 2018; 117,790 in 2019; 51,362 in 2020; and 42,891 in 2021. In 2022, the number of visits increased to 90,356. The described tendency was seen in all age groups (Figure 1).

**Figure 2:**
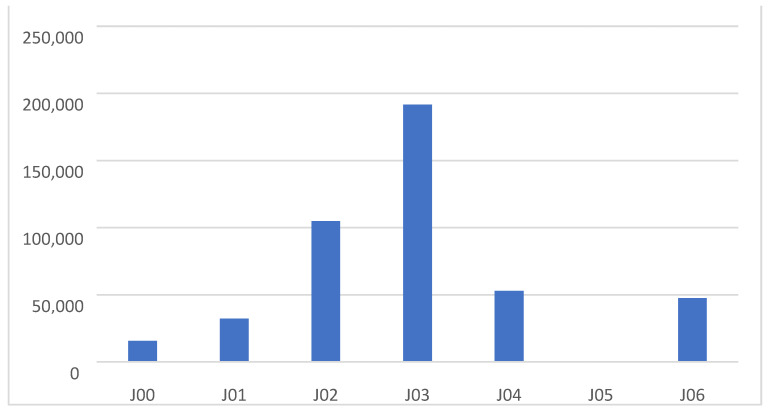
Number of URI diagnoses in the 2018–2022 period.

During the visits, acute nasopharyngitis (J00) was diagnosed in 3.50% of cases (*n* = 15,621), acute sinusitis (J01) in 7.25% (*n* = 32,292), acute pharyngitis (J02) in 23.57% (*n* = 104,953), acute tonsillitis (J03) in 43.01% (*n* = 191,580), acute laryngitis and tracheitis (J04) in 11.87% (*n*= 52,932), acute obstructive laryngitis [croup] and epiglottitis (J05) in 0.12% (*n* = 525), and acute upper respiratory infections of multiple and unspecified sites (J06) in 10.65% (*n* = 47,425) of cases (Figure 2).

**Figure 3:**
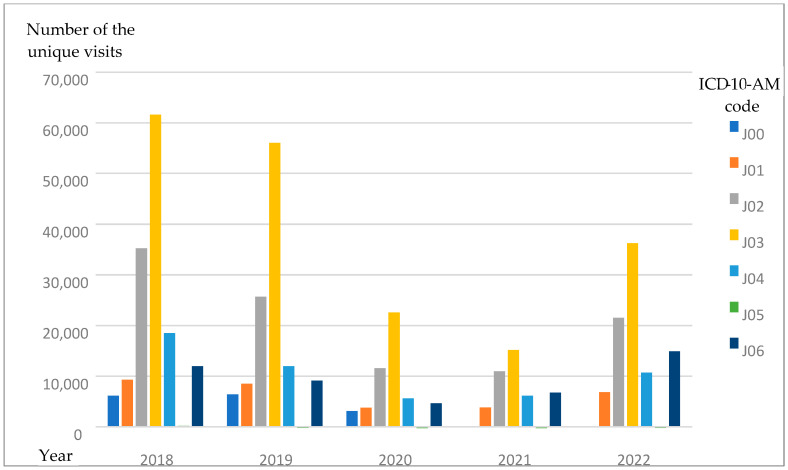
The number of URI diagnoses in different years during the 2018–2022 period.

**Figure 4:**
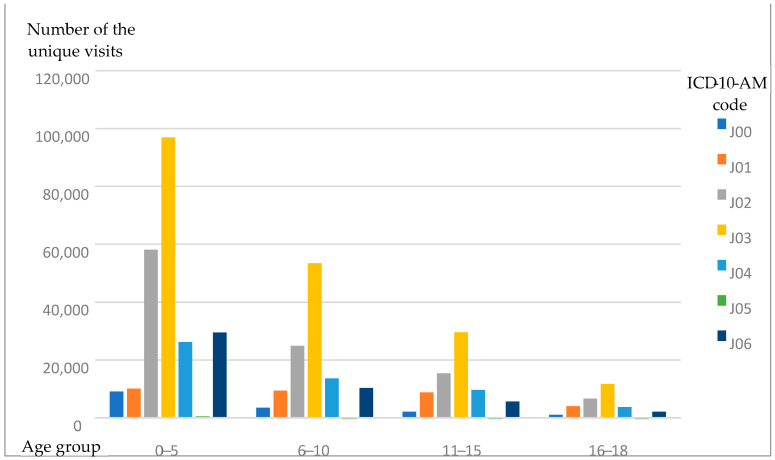
The number of URI diagnoses in different age groups during the 2018–2022 period.

The described tendency—with tonsilitis (J03) being the most popular diagnosis when antibiotics were prescribed, pharyngitis (J02) taking second place, and acute laryngitis and tracheitis taking third place—was seen throughout the entire analyzed period (Figure 3) and almost in all age groups (with the 0–5 age group being the exception, with acute upper respiratory infections of multiple and unspecified sites (J06) taking third place) (Figure 4).

**Figure 5:**
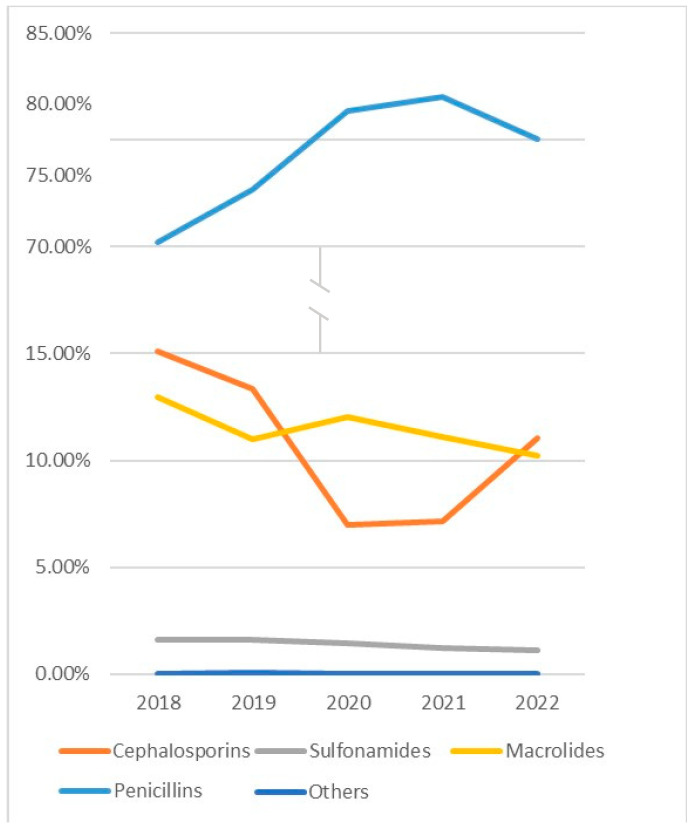
Proportions of antibiotic classes prescribed in the period of 2018–2022. Others include gentamicin, ciprofloxacin, metronidazole, nitrofurantoin, and doxycycline.

**Figure 6:**
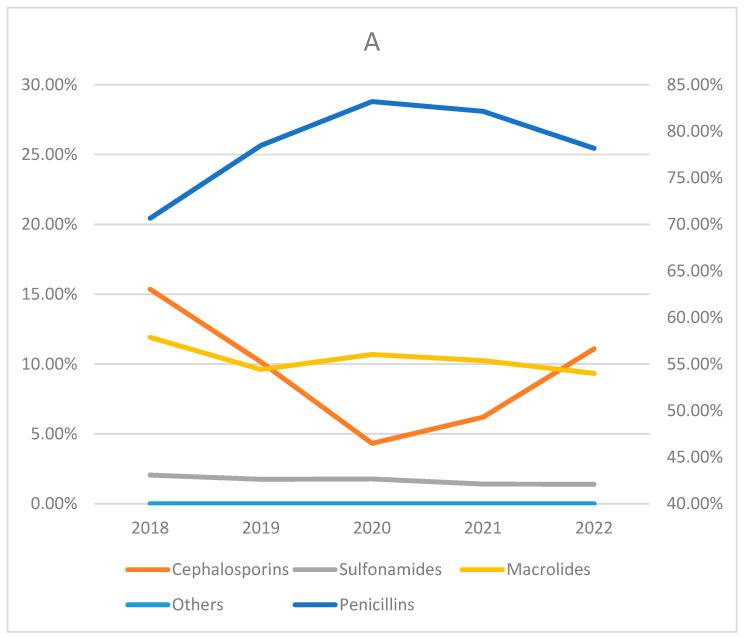

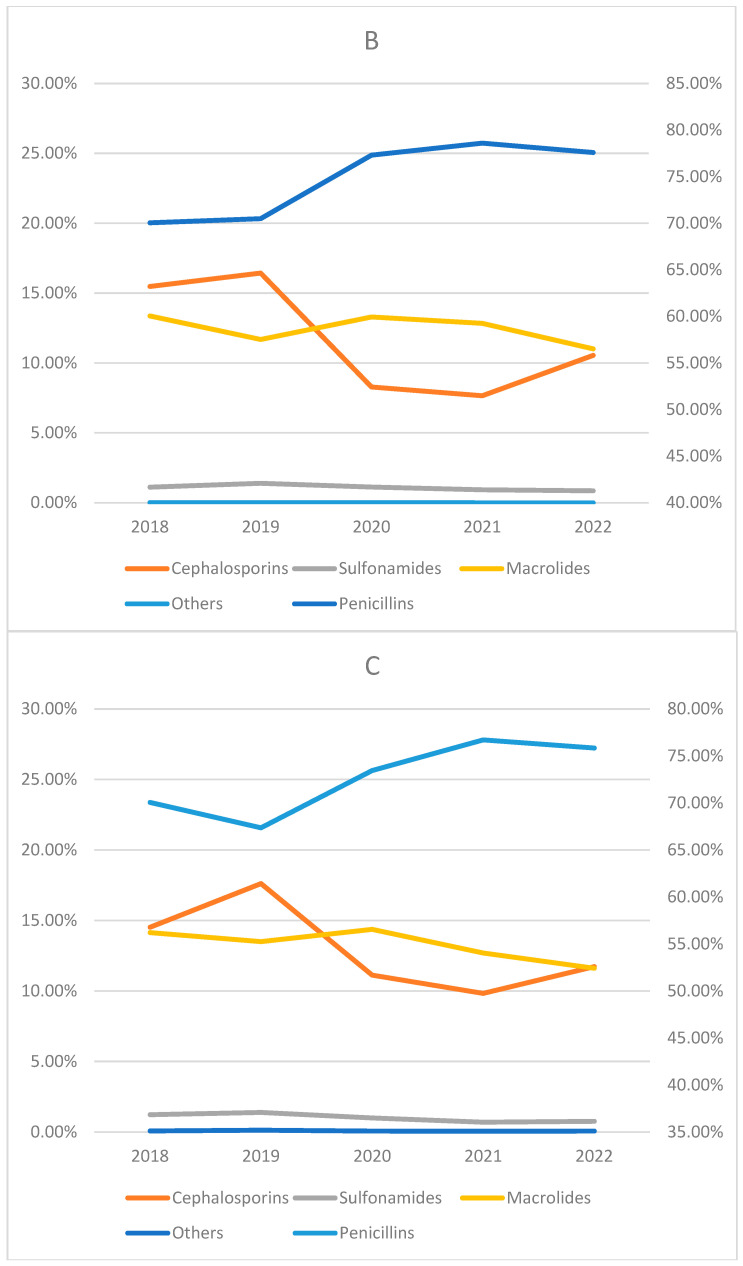

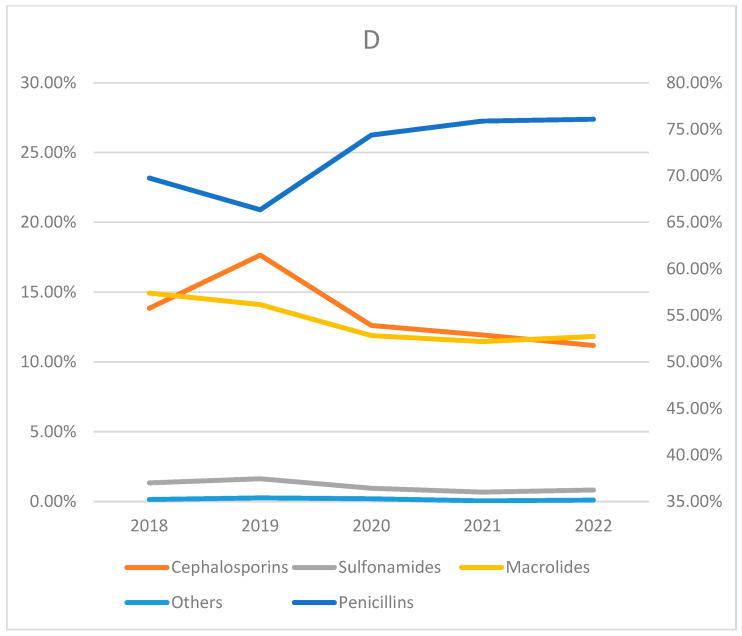
The proportions of antibiotic classes prescribed in the period of 2018–2022 between age groups. Others include gentamicin, ciprofloxacin, metronidazole, nitrofurantoin, and doxycycline. (**A**) The 0–5 age group; (**B**) the 6–10 age group; (**C**) the 11–15 age group; (**D**) the 16–18 age group. Percantage of the penicillins is shown on the right side. Percantage of all other antibiotic groups is shown on the left side.

Despite there being a decline in the number of absolute visits during the first four years of the analyzed period, the proportion of prescribed penicillin-class antibiotics increased: penicillins were prescribed in 70.30% of cases in 2018 when antibiotics were given to treat URIs and in 80.51% of cases in 2021, resulting in a 14.52% relative increase. In 2022, the proportion of prescribed penicillins decreased to 77.56%. The reverse dynamics were observed in cephalosporin prescribing practices: they were used in 15.11% of cases in 2018 and in 7.17% of cases in 2021, resulting in a 52.55% relative decrease. Then, the proportion of prescriptions increased to 11.05% in 2022. The proportion of prescriptions for macrolides and sulphonamides remained stable over the study period, averaging 11.47% and 1.38%, respectively, throughout the years (Figure 5). A similar trend was observed among all age groups analyzed (Figure 6).

Penicillins were mostly prescribed for the treatment of acute nasopharyngitis (in 79.31% of cases when antibiotics for this diagnosis were prescribed), and cepharlosporins were mostly used for acute tonsillitis (in 20.42% of cases when antibiotics for tonsillitis were prescribed). Macrolides were mostly used to treat acute laryngitis and tracheitis (34.08% of cases when antibiotics for this diagnosis were prescribed). The distributions of antibiotic classes by diagnosis in absolute numbers and percents for relevant disease code can be seen in Table 3.

Of all penicillin-class antibiotics, the most popular choice was amoxycillin (55.25% of cases when penicillins were prescribed). The primary choice of cephalosporin was cefadroxil (98.27% of cases when cephalosporins were prescribed), and the primary choice of macrolide was clarithromycin (81.96% when macrolides were prescribed). The absolute number of antibiotics prescribed for each diagnosis can be seen in Table 4.

## 3. Discussion

Our study showed that the largest proportion of antibiotics were given to children aged 0–5. This greatly correlates with other similar studies in which prescriptions of antibiotics for children were analyzed. As an example, in a retrospective cross-sectional study, Zhitong Zhang et al. analyzed 9340 prescriptions and found that the distribution among age groups was inconsistent: 7117 (76%) prescriptions were made for children aged 2–5, and 2223 (24%) for children aged 6–14 [18]. However, contrary to our study, where most antibiotics (43.01%) were used for the treatment of acute tonsillitis, in Zhitong Zhang et al.’s analysis, 92% of antibiotics were prescribed for the treatment of acute upper respiratory infections of multiple and unspecified sites. In addition to that, our analysis shows that the most popular antibiotic group was penicillins, whereas Zhitong Zhang et al. demonstrated the popularity of cephalosporins (66% of all cases). However, we must stress that in 2022, Lithuania and all of Europe faced a shortage of penicillins; therefore, a noticeable increase in cephalosporin usage could be seen in that year (Figure 5 and Figure 6).

In Sun Mi Shin et al.’s analysis, where data from Korea Health Insurance Review & Assessment Service was used, the tendency of prescribing antibiotics for younger age groups is also seen. Of all children treated with antibiotics, the 2–6 age group makes up 60.1% [19]. In their study, Sun Mi Shin et al. divided diagnoses into two categories: diseases when antibiotics are indicated and diseases when they are not indicated. In the authors’ opinion, antibiotics should be prescribed for acute sinusitis, acute pharyngitis, and acute tonsillitis. Diagnoses which do not require antibiotic therapy are acute nasopharyngitis, acute laryngitis and tracheitis, acute obstructive laryngitis croup and epiglottitis, and acute upper respiratory infections of multiple and unspecified sites. According to this division, their analysis concluded that in 67.3% of cases, antibiotics were prescribed when indicated, and in 41.9% of cases, antibiotics were not prescribed when indicated. If this division was used in our analysis, we could conclude that antibiotics were unnecessary in 26.14% of cases.

It can be seen in previously mentioned studies that the ratio of children diagnosed with URIs and children who were treated with antibiotics exceeds the WHO’s recommended standard of 20–26.8% [20]. This tendency is seen in other studies, too: the study by Takahiro Higashi and Shunichi Fukuhara, conducted in Japan, concluded that 60% of patients with URIs were treated with antibiotics [21]. Abdul-Nasiru Sumaila and Philip Teg-Nefaah Tabong noticed in a similar analysis that 28.6% of children with URIs under 5 years of age were prescribed antibiotics [22].

The situation may seem better in Western countries. In Norway, 26.2% of children with URIs were treated with antibiotics [23], and in USA, the proportion was 16% [24,25]. However, the assessment of antibiotic usage in Western countries within the framework of the World Health Organization (WHO) AWARE recommendations reveals a variety of trends. Studies such as those by Gerber et al. [26] and Williams et al. [27] demonstrated challenges in aligning pediatric antibiotic prescribing practices with global guidelines. While some Western nations have made progress regarding the principles advocated by WHO AWARE, there remains considerable variability in the rates of antibiotic prescriptions for pediatric URTIs, as demonstrated by Gerber et al. [26]. Factors such as parental expectations, diagnostic uncertainties, and concerns about potential bacterial co-infections contribute to this variability. The imperative for targeted interventions is noted in the work of Coxeter et al. [28], emphasizing the need for parental education and clear guidelines for healthcare professionals.

In Lithuania, a draft of National Recommendations on the Rational Use of Antibiotics was prepared for 2023, which is expected to help standardize antibiotic prescribing tactics in the country for the treatment of pediatric respiratory infections. The recommendations, for example, recommend phenoxymethylpenicillin as the first-line treatment for acute tonsillitis or tracheitis in children, leaving clarithromycin as the second-line choice. As can be seen from our study, current practices currently contradict these recommendations as amoxicillin is predominantly used to treat the diseases in question. The same situation is observed in the treatment of acute sinusitis—while guidelines recommend phenoxymethylpenicillin or amoxicillin as the first-line treatment, amoxicillin with clavulanic acid is the most popular in practice.

It is also evident in our study that absolute antibiotic prescribing practices decreased during COVID-19 lockdowns. This has been a notable phenomenon throughout Europe. Adlhoch et al. [29] provided plausible reasons for this. The implementation of public health measures, including social distancing, enhanced hygiene practices, and reduced community mobility, likely played a role in limiting the transmission of respiratory infections, both viral and bacterial. Consequently, healthcare providers were faced with a lower burden of URTIs, leading to a decrease in empirical antibiotic prescribing practices. However, Shmueli et al. [30] argued that the pandemic led to a dramatic increase in the use of healthcare services, changes in the circulation patterns of respiratory pathogens, a reduction in the number of non-respiratory infections, a disruption of routine childhood vaccination programs, and inappropriate use, as well as over-use, of antibiotics. Even though our study contradicts the latter statement, it is essential to acknowledge the potential dangers of decline in antibiotic usage for the treatment of URTIs, such as the risk of delayed antibiotic treatment for bacterial infections. As the world transitions out of the COVID-19 era, ongoing research and surveillance are critical to understanding the long-term implications of these changes in pediatric antibiotic usage patterns and ensuring a balanced approach to antibiotic prescribing practices in the post-pandemic landscape.

### Strengths and Limitations

The main advantage of this study is that the analyzed data were obtained from Lithuanian CHIF electronic records, which provided data on 445,328 visits with upper respiratory tract infections. However, the consumption of non-reimbursed medicines could not be included as this information is not available in CHIF records. In addition, our study could not provide information on the number of non-antibiotic visits in the years reported on. This poses limitations in determining the relative amounts of antibiotic prescriptions in a country. As we did not analyze patients’ medical records, which would have allowed us to analyze whether the patients’ complaints met the diagnostic criteria of the coded diagnoses and, consequently, to determine whether antibiotic therapy was justified, but we instead analyzed statistical extracts from the electronic records of the CHIF, our study can only provide a general picture of antibiotic consumption in the country during the period in question. In addition, our study does not reflect the exact durations of antibiotic treatment.

## 4. Materials and Methods

Data which were used in this observational study were collected from the CHIF’s electronic records. The CHIF collects information on prescriptions for medicines dispensed in all Lithuanian hospitals and primary care centers. The raw data were obtained from the CHIF upon the request of the authors.

The study population consisted of children aged between 0 and 18 years who visited their primary care doctors (pediatricians or family doctors) between January 2018 and December 2022 and were prescribed antibiotics for the treatment of URIs. The URIs in this study are diagnoses, which are coded as J00 (acute nasopharyngitis), J01 (acute sinusitis), J02 (acute pharyngitis), J03 (acute tonsillitis), J04 (acute laryngitis and tracheitis), J05 (acute obstructive laryngitis [croup] and epiglottitis), and J06 (acute upper respiratory infections of multiple and unspecified sites) in IC-10-AM (lit. TLK-10-AM).

The children were divided into four age groups: 0–5, 6–10, 11–15, and 16–18. The prescribed antibiotics in the electronic records were classified using the Anatomical Therapeutic Chemical Classification (ATC) system.

### 4.1. Statistical Analysis

All children with URIs’ unique visits to primary care doctors were included in calculations. A unique visit is defined as a single visit to a doctor by a unique patient when an antibiotic is prescribed, i.e., repeat visits where the doctor follows the patient’s treatment are excluded from the statistical analysis. SPSS statistical software package version 26.0 (IBM/SPSS, Inc., Chicago, IL, USA) and Microsoft Excel 2016 were used for the data management and calculations. Absolute figures are given for the number of visits, diagnoses, and specific antibiotics prescribed. Relative figures are given to describe trends in prescribed classes of antibiotics.

### 4.2. Ethics

Informed consent was not acquired from patients nor their parents or guardians because this was a large register-based study, and information from which individual persons could be identified was not collected. Anonymized data were provided to the investigators by the CHIF at the request of the Children’s Disease Clinic of the Vilnius University Faculty of Medicine. Data were received, processed, and stored in accordance with the General Data Protection Requirements (GDPR).

## 5. Conclusions

Our study demonstrates that during the period of 2018–2022, the number of prescriptions for antibiotics for URTIs decreased, but prescriptions for penicillin-class antibiotics increased in a relative manner. The most common diagnoses during these visits were acute tonsillitis and acute pharyngitis, and most antibiotic prescriptions were for children in the 0–5 age group. If Lithuania’s National Recommendations on the Rational Use of Antibiotics were implemented during the analyzed period, the antibiotic prescribing tendencies would not meet them. Therefore, in the future, an analogous study could assess whether antibiotic prescribing trends have changed since the introduction of the guidelines in 2023.

## Figures and Tables

**Table 1:** Unique visits by age group in the 2018–2022 period when antibiotics were prescribed to treat URIs. The proportion of patients by age group in the given year is expressed as a percentage in brackets.

	Year	2018	2019	2020	2021	2022	Total
Age Group							
0–5		65,287 (45.68)	62,740 (53.26)	26,601 (51.80)	27,362 (63.80)	48,227 (53.37)	230,217 (51.70)
6–10		39,420 (27.58)	30,127 (25.58)	12,933 (25.18)	8481 (19.77)	24,086 (26.66)	115,047 (25.83)
11–15		25,979 (18.18)	17,621 (14.96)	8243 (16.05)	5137 (11.98)	14,026 (15.52)	71,006 (15.94)
16–18		12,243 (08.57)	7302 (06.20)	3585 (06.98)	1911 (04.46)	4017 (04.45)	29,058 (06.53)
Total		142,929 (100)	117,790 (100)	51,362 (100)	42,891 (100)	90,356 (100)	445,328 (100)

**Table 2:** The number of unique antibiotic prescribing visits per capita in a given year for a given group of children during the 2018–2022 period.

	Year	2018	2019	2020	2021	2022	Total
Age Group							
0–5		0.38	0.37	0.16	0.17	0.31	0.28
6–10		0.28	0.21	0.09	0.06	0.17	0.16
11–15		0.20	0.14	0.06	0.04	0.10	0.11
16–18		0.14	0.09	0.05	0.02	0.05	0.07
Total		0.27	0.22	0.10	0.08	0.17	0.17

**Table 3:** The distribution of antibiotic classes by diagnosis in absolute numbers and percentages in the period of 2018–2022. Others include gentamicin, ciprofloxacin, metronidazole, nitrofurantoin, and doxycycline.

	Diagnoses	J00	J01	J02	J03	J04	J05	J06	Total
Antibiotics									
Penicillins		12,389 (79.31)	25,489 (78.93)	85,313 (81.29)	139,911 (73.03)	32,142 (60.72)	368 (70.10)	37,536 (79.15)	333,148 (74.80)
Cephalosporins		501 (3.21)	830 (2.57)	10,295 (9.81)	39,121 (20.42)	1110 (2.10)	18 (3.43)	2075 (4.38)	53,950 (12.11)
Sulfonamides		605 (3.87)	323 (1.00)	1803 (1.72)	508 (0.27)	1617 (3.05)	4 (0.76)	1543 (3.25)	6403 (0.01)
Macrolides		2122 (13.58)	5620 (17.40)	7521 (7.17)	12,010 (6.27)	18,039 (34.08)	135 (25.71)	6254 (13.19)	51,701 (11.60)
Others		4 (0.03)	30 (0.09)	21 (0.02)	30 (0.02)	24 (0.05)	0 (0)	17 (0.04)	126 (0.0)
Total		15,621 (100)	32,292 (100)	104,953 (100)	191,580 (100)	52,932 (100)	525 (100)	47,425 (100)	445,328

**Table 4:** The absolute number and percentage of antibiotics prescribed for each diagnosis. Amx—amoxycillin; Pcv—phenoxymethylpenicillin; Amc—amoxicillin and clavulanic acid; Stm—sultamicillin; Cfr—cefadroxil; Cxm—cefuroxime; Sxt—sulfamethoxazole and trimethoprim; Ery—erythromycin; Clr—clarithromycin; Azm—azithromycin; Dox—doxycycline. Others include ciprofloxacin, spiramycin, gentamicin, cefprozil, metronidazole, and nitrofurantoin.

	Diagnoses	J00	J01	J02	J03	J04	J05	J06	Total
Antibiotics									
Amx		7391 (47.31)	10,870 (33.66)	58,232 (55.48)	58,238 (30.40)	21,932 (41.43)	213 (40.57)	27,184 (57.32)	184,060 (41.33)
Pcv		1318 (8.44)	517 (1.60)	14,598 (13.91)	53,804 (28.08)	1519 (2.87)	59 (11.24)	3319 (7.00)	75,134 (16.87)
Amc		3213 (20.57)	13,189 (40.84)	10,829 (10.32)	24,216 (12.64)	7851 (14.83)	86 (16.38)	6444 (13.59)	65,828 (14.78)
Stm		467 (2.99)	913 (2.83)	1654 (1.58)	3653 (1.91)	840 (1.59)	10 (1.90)	589 (1.24)	8126 (0.02)
Cfr		492 (3.15)	747 (2.31)	10,195 (9.71)	38,483 (20.09)	1078 (2.04)	18 (3.43)	2002 (4.22)	53,015 (11.90)
Cxm		8 (0.05)	78 (0.24)	100 (0.10)	633 (0.33)	32 (0.06)	0 (0)	73 (0.15)	924 (0.00)
Sxt		605 (3.87)	323 (1.00)	1803 (1.72)	508 (0.27)	1617 (3.05)	4 (0.76)	1543 (3.25)	6403 (0.01)
Ery		4 (0.03)	6 (0.02)	79 (0.08)	98 (0.05)	104 (0.20)	0 (0)	14 (0.03)	305 (0.0)
Clr		1808 (11.57)	4269 (13.22)	6066 (5.78)	10,307 (5.38)	14,493 (27.38)	85 (16.19)	5345 (11.27)	42,373 (0.10)
Azm		310 (1.98)	1345 (4.17)	1375 (1.31)	1605 (0.84)	3442 (6.50)	50 (9.52)	895 (1.89)	9022 (0.02)
Dox		3 (0.02)	17 (0.05)	18 (0.02)	17 (0.01)	18 (0.03)	0 (0)	11 (0.02)	84 (0.0)
Others		2 (0.01)	18 (0.06)	4 (0)	18 (0.01)	6 (0.01)	0 (0)	6 (0.01)	54 (0.00)
Total		15,621 (100)	32,292 (100)	104,953 (100)	191,580 (100)	52,932 (100)	525 (100)	47,425 (100)	445,328

## Data Availability

Inquiries can be directed to the corresponding author.
